# The perspectives of Swedish registered nurses about managing difficult calls to emergency medical dispatch centres: a qualitative descriptive study

**DOI:** 10.1186/s12912-021-00657-5

**Published:** 2021-08-18

**Authors:** Inger K. Holmström, Elenor Kaminsky, Ylva Lindberg, Douglas Spangler, Ulrika Winblad

**Affiliations:** 1 School of Health, Care and Social Welfare, Mälardalen University, Västerås, Sweden; 2 Department of Public Health and Caring Sciences, Uppsala University, Uppsala, Sweden

**Keywords:** Emergency calls, Emergency medical dispatch centres, Interviews, Nursing, Systematic text condensation

## Abstract

**Background:**

Telephone triage at emergency medical dispatch centres is often challenging for registered nurses due to lack of visual cues, lack of knowledge about the patient, and time pressure – and making the right decision can be a matter of life and death. Some calls may be more difficult to handle, and more knowledge is needed about these calls to develop education and coping strategies. Therefore, the aim of this study was to describe the perspectives of registered nurses’ views about managing difficult calls to emergency medical dispatch centres.

**Methods:**

A descriptive design with a qualitative inductive approach was used. Three dispatch centers in mid-Sweden were investigated, covering about 950,000 inhabitants and handling around 114,000 calls per year. Individual interviews were carried out with a purposeful sample of 24 registered nurses. Systematic text condensation was conducted.

**Results:**

Seven themes were generated: *calls with communication barriers, calls from agitated or rude callers, calls about psychiatric illness, calls from third parties, calls about rare or unclear situations, calls with unknown addresses* and *calls regarding immediate life-threatening conditions*. There was a strong consensus among the registered nurses about which calls were experienced as difficult, with the exception of calls about immediate life-threatening conditions. Some registered nurses thought calls about immediate life-threatening conditions were easy to handle as they simply adhered to protocol, while others described these calls as difficult and were emotionally affected.

**Conclusion:**

The registered nurses’ descriptions of difficult calls focused on the callers, while their own role, the organisational framework, and leadership were not mentioned. Many types of calls included difficulties, which could be related to the caller, their symptoms, or different circumstances. The registered nurses pointed to language barriers and rude, agitated callers as increasing problems. An investigation of actual emergency calls is warranted to examine the extent and nature of such calls.

## Background

During recent decades, an increased international demand for emergency services has been reported in many countries [1, 2]. This may be due to easier accessibility as the majority of citizens have cell phones, but also to an increasing worry and anxiety in the population [3, 4]. To aid citizens’ contact with healthcare services, telephone nursing and triage have been established as integral parts of healthcare in many countries such as the UK, the US, Canada, and Australia [5]. In Sweden, telephone triage is mainly carried out within three contexts: at primary healthcare centres, at the national telephone nursing service Swedish Healthcare Direct (SHD) and at emergency medical dispatch centres (EMDCs). EMDCs take calls with a higher degree of urgency, including immediate threats to a patient’s life. At EMDCs, triage calls are completed either by registered nurses or non-nurse dispatchers in the primary care-taking role. For the present study, nurses from three emergency dispatch centres staffed exclusively by nurses were interviewed. The communication in EMDC calls has been reported as difficult [6]. Therefore, we sought to dig deeper into nurses’ actual experience of difficulty in such calls. If the difficulties can be pinpointed, strategies can be developed, and nurses trained to provide optimal handling of these calls. Hence, much can be learned from nurses’ experiences of what they find as difficult in EMDC calls.

A recent review [7] suggests that EMDC telephone triage takes place in three steps: identifying the event, assessing the caller’s need for support, and prioritizing the response. The dispatcher (in this case a registered nurse) asks questions to identify care needs and analyse the emergency situation, considers sending ambulance assistance to the correct address, and assess how urgent the illness / accident is to decide how fast this should be carried out. Nurses are expected to handle EMDC calls by asking questions, and callers are expected to answer. The nurse often provides concrete first aid and emergency response instructions during the call. The advice given in EMDC calls is more steering, direct, concrete and forceful than in other telephone triage contexts [8]. Sometimes however, callers resist or reject the nurses questioning [8], and may view the questions as irrelevant, believing that the emergency help they need is being delayed [9].

The process of telephone triage is challenging for nurses due to a lack of visual cues, lack of knowledge about the patient, and time pressure [10–12]. Triage in general may also include challenges such as linguistic barriers, cultural differences, personal perceptions, or environmental impacts [13, 14]. Callers in general want to be listened to, taken seriously, to tell their story without interruption, and be treated in an empathic way [15, 16]. Nurses, on the other hand, expect callers to provide accurate information and clear answers to their questions, and that callers trust them in posing the right questions and making credible and competent triage decisions [8]. Public expectations of telephone triage are dominated by the need for trustworthy, helpful interactions [3], handling of clinical problems, effective management and triage decisions [17]. A literature review has shown that trust in the nurse-patient relationship is essential to establish in encounters, also short EMDC calls, and nurses professional competencies and interpersonal caring attributes are important in developing trust [18]. Theoretically, trust generates a context in which patients give valid and reliable information [19]. The caller should be able to trust the healthcare system, the nurses’ competence, and their will to provide optimal care and treatment.

Telephone triage in EMDC calls is even more complex than in non-urgent telephone triage, as the level of urgency is higher, there is more time pressure, and the decisions made are often a matter of life and death [8, 14, 20]. Furthermore, the fear of making wrong decision in emergency calls is always in the back of nurses’ heads [21], especially as calls need to be kept short and concise to increase accessibility. Many calls are made by a third party, such as a family member, a friend or sometimes a passing stranger, which makes the situation even more complicated.

Some calls are stated to be more difficult than others. Lindström et al [20] report that challenges appeared in callers' descriptions of unclear symptoms, paradoxes, and the nurses’ lack of communication strategies during the EMDC call. In addition, reaching a mutual understanding between nurse and the caller is described as the utmost challenge for nurses at EMDCs [22].

To support emergency call triage, nurses commonly employ clinical decision support systems (CDSS). These are often "expert systems" which consists of a set of predetermined assessment rules. A recent review paper concluded that the success of emergency telephone triage is dependent on using a well-established protocol, supported by a CDSS and continuing training for staff [7]. A CDSS can provide a structure for calls, improve correct decision-making and hence make assessments safer [23–25]. Nurses at EMDCs, however encounter a range of more or less complex situations and persons in crisis, where the computerized CDSS and internet-access might only provide help to a certain extent. For that reason, they also need to rely on professional competence and experience and might develop strategies to deal with such difficult calls and situations.

Taken together, EMDC triage includes many challenges, most of which seems to be related to communication. To date, we know that some calls are more difficult than others. However, most studies of telephone triage are from non-urgent settings or focus on dispatchers without a nursing degree and as such, studies of nurses’ work in emergency medical dispatch are rather scarce. More knowledge is thus needed about the nature and characteristics of difficult EMDC calls, and of how nurses experience such calls. With better knowledge, education and training programmes can be developed to help nurses better prepare for such calls. Therefore, the aim of this study was to describe the perspectives of registered nurses’ views about managing difficult calls to emergency medical dispatch centres.

## Methods

### Design

The study had a qualitative descriptive design inductive approach for data analysis [25]. This approach was appropriate to map the perspectives and experiences of the nurses in an area with limited published knowledge.

### Sample and setting

Three EMDCs in mid-Sweden, covering about 950,000 inhabitants, were included in the study. The EMDCs were open 365/24/7 and handle around 114,000 calls yearly. From these three centres, a purposeful sample of 24 nurses were interviewed. To get rich and diverse data, we aimed obtain a diverse sample including nurses with long and short working experience, and of different age and gender. Nine males and 15 females between 34 and 64 years old, with working experience ranging from 5 to 44 years were interviewed. Eleven of the nurses had one or more specialist educations (60 ETCS) in addition to their three-year bachelor’s degree in nursing.

### Data collection

Four of the authors made informal observations of nurses work at two of the EMDCs. This was done to get acquainted with the nurses’ work and setting and to enable posing more initiated interview questions. The informal observations were not part of the present dataset. Thereafter, three pilot interviews were performed by the second author to test the interview guide. This led to a reduction of the interview guide to focus the interviews. The pilot interviews were however rich in content and were included in the dataset and analysed together with the subsequent interviews. The interviews began with background questions the professional background and education of the nurses and progressed to questions about their work processes and professional practice. The interview guide contained questions such as:

1. What is the core of the EMDC work in your view?
2. When do you feel that you have success in your EMDC work?
3. What is difficult or what hinders you in your EMDC work?
4. Are there any special types of calls which are more challenging or difficult?
5. What are the pros of the CDSS?
6. What are the development areas of the CDSS?
7. Please describe a case when the CDSS was helpful.
8. Please describe a case when the CDSS was not helpful.

Probing questions such as: *please give a concrete example* and *please tell me more* were used to make the nurses elaborate on their answers, if needed. The findings about CDSS use are reported elsewhere [26].

The managers of the three included EMDC-sites were contacted and asked to recruit seven nurses each. Nurses at each site were requested to participate in the study via mailing list, and additional nurses were recruited via direct contact by the managers at the respective EMDC sites. The managers were requested to provide a diverse sample of nurses to interview.

A research assistant with both experience as an nurse and qualitative research methods performed the interviews. The interviews were conducted between March 2018 and October 2019. They took place in a private room at the respective EMDCs, except for one which was performed by telephone. The interviews ranged from 19 to 57 minutes (mean 37 minutes). After 15 interviews, data started to become saturated, but as we wanted to include the same number of nurses from each EMDC site, interviewing continued.

### Data analysis

The interviews were transcribed verbatim and processed as text. A thematic analysis using systematic text condensation (STC), was conducted according to Malterud [27]. STC is a pragmatic analytical procedure, inspired by the steps in Giorgi's phenomenological method (see e.g. [28]). The analysis was carried out in four steps. Step 1 included getting an overall impression of the data and identifying preliminary themes. Step 2 entailed the identification and sorting of meaning units arising from the preliminary themes. This coding was done by the first author who identified, classified, and sorted meaning units potentially related to the preliminary themes. Step 3 included condensation, which involved having meaning units de-contextualized and classified into subgroups. Preliminary theme labels were then set, reviewed, and discussed by all authors. Step 4 consisted of a synthesis of the results. The subgroups were reviewed and a narrative synthesis was written as an analytic text, including quotations as illustrative examples. The analysis was repeatedly discussed at team meetings with all authors, and disagreements were settled by negotiated consensus. The COREQ- checklist was used to assure quality [29], see appendix 1.

### Results

Seven themes regarding registered nurses’ experiences of difficult calls to emergency medical dispatch centres were generated: *calls with communication barriers, calls from agitated or rude callers, calls about psychiatric illness, calls from third parties, calls about rare or unclear situations, calls with unknown address* and *calls regarding immediate life-threatening conditions*. The themes are presented below with illustrative quotes.

### Calls with communication barriers

A recurring theme was calls with communication barriers. Communication barriers could have a number of different causes. Most commonly mentioned was a *lack of ability to speak and understand Swedish*, which is common as Sweden increasingly is a multicultural country with many immigrants.

*“Language barriers have increased…as there are all the more immigrants in our society. // Some shifts there are like 80 % of calls made by someone who is not fluent in Swedish”* [11]

The caller not being fluent in Swedish was considered sub-optimal from a patient safety perspective and made assessments harder. Other communication barriers were the caller *having impairment in hearing and speaking*, like aphasia. Yet another communication barrier could be if the caller was *under the influence of alcohol or drugs*, which made their talk thick and hard to grasp. A further factor affecting communication was *the telephone connection* and for instance weather conditions with disturbing background noise which made it hard to pick up the caller’s speech. Despite the different origins, all these situations created problems for the nurses in getting the information they needed from the caller, and problems making themselves understood.

*“If Swedish isn’t their mother tongue, or if they’re aphasic or really drunk*. *Or they could have some kind of very difficult speech impediment. Then communication is negatively affected ‘cause I cannot understand them”* [13]

### Calls from agitated or rude callers

Agitated callers were frequently mentioned as difficult to handle and reported to be an increasing problem. The agitation could manifest in different ways. Some callers were *screaming due to fear, pain, and panic* as they found themselves in a difficult and dramatic situation which they had no experience of. They might be ill or injured themselves, or a family member or friend could be affected:

*“And then there are “the yellers”. I had a call from a young girl who just yelled the whole call through. The only thing I could grasp that her mother was ill, and then I couldn’t get more out of her. I got the address, and she kept yelling “send an ambulance” and a whole lot of nasty things”* [14]

Other callers were *rude and angry* and could use bad language or even threaten the nurse. According to the nurses, callers sometimes expressed that nurses should just do what they are told (i.e. send an ambulance) without asking further questions, and did not understand the organization, prioritizing and recourses available in acute and emergency care. In such cases the caller might refuse to answer the nurses’ questions:

*“…a very agitated man…I did not consider his condition as critical, but it’s hard indeed when the caller choses to not answer my questions regardless of being capable to do so* [4]*.*

Agitated callers could stress the nurses, making them feel to be under attack and questioned in their professional role. At worst, nurses became agitated themselves, which in turn made communication even harder. Being threatened and yelled at could also lead to the nurse abstaining from asking further questions.

*“those who are aggressive if they don’t get their requested ambulance, and they don’t listen when I ask my questions to get information and help them”* [18]

### Calls about psychiatric illness

Callers with *psychiatric illnesses,* like severe anxiety, depression, and suicidal thoughts, were considered more vulnerable and harder to assess and provide adequate help and nursing care for. Sometimes these calls could start with an upset caller presenting with breathing problems and chest pain, and it could be hard to rule out if the symptoms were of somatic or psychiatric origin. In other cases, there were relatives calling for patients with psychiatric illnesses. These relatives could have come to a breaking point when they could not handle things on their own. However, in these cases the patients themselves could refuse getting help, and the ambulance staff could not force their care on the patient.

*“…threatening to take their own life and describe that life is without meaning. // a feeling of not getting help, isolation, loneliness and with severe anxiety. // being out in a forest with the intention of suicide”* [21]

The nurses pointed to that none of them had a specialist education in psychiatric care, and they felt a lack of competence when handling calls from vulnerable persons with psychiatric symptoms. When they talked to someone amid a suicide attempt, it was described as like walking on very thin ice. The nurses emphasized that every word they said had to be considered with care in these cases, otherwise the consequences could be fatal. Simultaneously, they needed to act instantaneously to avoid a fatal outcome. They were emotionally affected, especially if the call was about a child or teenager:

*“We had three hangings in eight days, two were 21 and one just 11…”* [11]

In connection to this, nurses also mentioned *frequent callers* who often called several times a week, and who could have different types of psychiatric and somatic problems and be worried and lonely.

“*Then there are the frequent callers, who call and want us to send an ambulance. Most often we know that there’s no need for an ambulance. This could be like [sighs] an obstruction…well not obstruction, but kind of difficult call”* [14]

A further complication could be if the caller had *both somatic and psychiatric problems*, and it was hard to rule out what was going on and were they could get optimal care. This was often the case with frequent callers.

### Calls from third parties

A common difficulty was calls made by a third party. The reasons for third-party calls were numerous. The most common third-party caller was a parent of a sick child. Parents were described as having differing capabilities in their ability to describe their child’s condition. Other third-party callers were adult children calling on behalf of an old parent, or a family member calling for someone who did not speak Swedish very well. Sometimes the third-party caller was not at the same location as the patient. They could be in another town but called after having talked to the patient e.g. an elderly parent. This made the nurses assessment difficult, as many of their questions were left unanswered. In some cases, the nurses had to speak to a young child, who might be the only person in the family to speak Swedish.

In the case of third party-callers, the nurses got the caller’s interpretation of the symptoms as the basis for their assessment, which made it much harder to make a correct triage decision. In cases of sudden illness or accidents, a passing stranger who did not have any background information about the ill person, could be making the call. All these situations made it harder to establish the severity of the symptoms.

*“The biggest difficulty is that sometimes I don’t get to speak to the patient in need. I talk to a relative or a stranger who could be incredibly stressed // I need to get answer to my questions to make a reliable assessment”* [10]

### Calls about rare or unclear situations

Rare situations were for instance drowning, electric shock, and suicides like hanging as well as obstetric conditions and childbirth. These *occurred very seldomly*, and the nurse might never have encountered such a case before:

*“Miscarriage…generally ob./gyn is not my best professional asset”* [2]

The nurses underscored that such calls were difficult to handle. In some cases, there was no search-term in the CDSS for the problem in question. The nurses could lack experience and competence in how to handle a rare situation, and hence receive no support from the CDSS, but was left in a vacuum. A further situation was when *the situation was unclear,* and the caller could not describe symptoms or what had happened. This made it difficult for nurses to pinpoint what was going on and the severity of the situation.

*“These patients who cannot explain clearly what’s wrong. // It’s something, they don’t feel well but cannot verbalize it clearly and I cannot, despite my professional experience and the CDSS pinpoint it. But I register that something’s wrong.”* [10]

Sometime there were also *doubts about the truth* of the caller’s description. The nurses described that people both exaggerate and underestimate their symptoms, or don’t provide the full picture.

*“If I don’t get any clear picture…the caller is vague in the symptom description, and I have difficulties grasping what they mean. // Bizarre really, my college thought that the caller had stomach ace “like a knife in my belly” when in fact the caller had a real knife in his stomach”* [5]

### Calls with unknown address

Getting the correct address is pivotal if an ambulance is to be sent out. Therefore, the nurses asked callers for the address early in the calls. Sometimes however, callers failed to report the current address, creating difficulties for the nurse. This could be due to *communication barriers*, as mentioned above. It could also be due to *stress and agitation* on behalf of the caller. There were however occasions when the callers are fully able to communicate but *do not know where they are*. This happened most often when callers were outdoors and hiking, skiing or horseback riding in new surroundings. It could also happen after stopping in a car, and the nurse would then ask the caller to describe the surroundings, and for instance what villages that they had passed to follow along on the map. If the caller was lost in a city or village and needed emergency help, they could often ask a passing stranger where they were and get help from them.

*“The caller doesn’t know where s/he is, like out in a forest picking mushrooms.”* [21]

### Calls regarding immediate life-threatening conditions

Some nurses stated that calls regarding immediate life-threatening conditions were experienced as difficult and stressful. However, this theme was the only one without consensus among interviewed nurses. While some nurses just turned to protocol and found these calls straightforward, other were emotionally affected and found the calls hard to handle.

*“ If it’s kids who are severely ill or cardiac arrest, then it’s hard to stay focused.”* [16]

If the patient was neither awake, breathing nor having a free airway, the nurses had only a few minutes to act to potentially save a life, and hence clear communication with the caller was paramount.

*“ these high priority calls about cardiac arrest, drowning, suffocation, hanging…”* [11]

Other nurses however explicitly did not experience these types of calls as difficult. They saw it as simple to just turned to the protocol on for instance instructing in CPR (cardiopulmonary resuscitation) and to follow it step by step. This degree of difference between nurses was not expressed in the other themes.

## Discussion

There was a strong consensus among the participating nurses about the calls perceived as difficult. The only exception was regarding immediate life-threatening conditions that only some of the nurses considered as challenging, as they got emotionally involved. Other nurses however just turned to the CDSS and followed the protocol and found these calls to be rather clear-cut. According to the participating nurses, calls which included language barriers and calls from rude or agitated callers have seemed to increase. Language barrier-related problems might be due to the increasing number of immigrants [30] with other native languages than Swedish, which calls for a need to implement fast-track interpreting services connected to healthcare lines, to aid the nurses with callers that are not fluent in Swedish. However, Jones [31] found that while language barriers could be a hindrance to developing trust in the patient-professional relationship, the nurse’s personality and way of being was more important. Hence, they concluded that patients are likely to develop trust also with nurses who do not speak their native language. These results call for further quantitative studies to establish the magnitude of such problems. Language barriers however increase time to dispatch and the accuracy of the level of aid provided during medical emergency calls [32]. Therefore, Meischke et al [32] suggest that decreasing the time to connecting to an interpreter when using an interpretation service could minimize delays. However, callers with speech problems, or intoxicated callers whose speech is hard to grasp, are not helped by an interpreter.

It was also evident from the interviews that the nurses experienced the difficulties as owing to the callers/patients in some way. Few of the nurses reflected on their own competence, communication or actions as part of making calls difficult. There were also few reflections about the organizational frames, working conditions, leadership and further education and training. The nurses did however consider the CDSS as a helpful tool [26]. According to Donabedian [33] quality of care depends on three aspects: structure, process and outcomes. To achieve optimal outcomes, both the caring processes (here the nurses communication and triage of callers’ needs) and the structure and organization in which they work in are essential. These underlying structural aspects would thus be interesting to investigate further.

In comparison with Higgins et al. [14], a study from the UK published nearly 20 years ago, we found both similarities and differences: they found that upset callers, language problems, vague descriptions and paediatric calls were problematic to handle. They also highlighted problems of getting the right location and address, which is pivotal in ambulance dispatch. In contrast with Hedman [8], the present nurses did not mention calls about domestic violence and abuse as difficult to handle.

However, in the Higgins et al. [14] study of 1.830 calls, only two callers were verbally abusive. In our interview data, this was stated to be a difficulty which prolonged the questioning-phase, i.e. delayed help to the afflicted person and created stressful situations for nurses. There seem to be an increasing trend of increasing assaults and threats to personnel in emergency services in general, which is also reported in Swedish media [34, 35]. Abraham et al [36] reported violence as one of the most stressful situations to handle for emergency department staff, and patients and relatives can react aggressively when stress and traumas occur [37]. The present nurses did not encounter physical violence, but rather verbal threats and abuse. Hedman [8] did not report of such verbal abuse, but found in his study of emergency calls that callers sometimes questioned or rejected nurses’ line of questioning, as they just wanted an ambulance to be sent out with no further discussion. Such a power struggle is contra-productive and delays the triage process [38]. The increase in verbal and physical abuse might potentially originate from a lack of trust in healthcare and its professionals. Trust in society as a whole is lower among unemployed, persons with ill health and those borne abroad [35], a pattern which might mirror their trust in emergency care services. Further research is certainly needed within this field.

The occurrence of communication problems related to the emotional state of the caller highlights the need to train nurses in dealing with people in emotional states [14]. Some callers do not seem to realise that there are a limited number of ambulances and resources available, and that the nurses have a crucial role in prioritising them in a safe and secure manner.

Our findings resembles some of the findings of Lindström et al [20], for instance that vague descriptions and communication problems were barriers in EMDC calls. A Danish study [6] showed that age, ethnicity, day of week and time of day were significant predictors of emergency call categorization as "unclear problem". About 19% of calls in the Copenhagen area were categorised as “unclear problem” [39]. When calls were categorized as an unclear problem, a higher mortality was observed. This suggests that obtaining a clear picture of the problem from the caller could make the difference between life and death.

Further research is warranted on nurses’ strategies for handling difficult calls, as well as investigation of the actual communication in EMDC calls.

### Strengths and limitations

Semi structured interviews and systematic text condensation [27] were deemed suitable for the present study aim. The sample was purposeful and had satisfactory information power [40] and was rich in experiential content with detailed descriptions. Rather than investigate specific actual calls to EMDCs, we were interested in general perspectives of nurses about managing difficult calls based on their experiences. The research group included both clinicians and non-clinicians which allowed for both emic and etic perspectives. As with all qualitative studies, the findings should prove transferrable to similar settings and contexts, but conclusions cannot be drawn regarding the actual prevalence of the types of calls we identify in this study.

## Conclusion

There was a strong consensus among nurses regarding which calls were difficult to handle at EMDCs, with the exception of calls regarding immediate life-threatening conditions which some nurses reported as being simple to deal with. In addition, the nurses most often viewed the difficulties in relationship to the callers in different ways, such as language barriers, agitation, or rudeness. These difficulties were stated to be increasing. At worst, such difficult calls might lead to incorrect triage decisions which could hamper patient safety and appropriate resource allocation. Further quantitative studies are needed to establish the magnitude of these problems, as well as studies of nurses’ strategies for handling them.

## Data Availability

The datasets generated and/or analyzed during the current study are not publicly available due to ethical reasons and the right to confidentiality for interviewed persons. Please contact the first author should you request data.

## Abbreviations

CDSS: Clinical decision support systems
COREQ: Consolidated criteria for reporting qualitative research
CPR: Cardiopulmonary resuscitation
EMDC: Emergency medical dispatch centre
ETCS: European Credit Transfer and Accumulation System
SDH: Swedish Healthcare Direct
STC: Systematic text condensation
